# Roadless Wilderness Area Determines Forest Elephant Movements in the Congo Basin

**DOI:** 10.1371/journal.pone.0003546

**Published:** 2008-10-28

**Authors:** Stephen Blake, Sharon L. Deem, Samantha Strindberg, Fiona Maisels, Ludovic Momont, Inogwabini-Bila Isia, Iain Douglas-Hamilton, William B. Karesh, Michael D. Kock

**Affiliations:** 1 Wildlife Conservation Society, Bronx, New York, United States of America; 2 Department of Biology and Whitney R. Harris World Ecology Center, University of Missouri - St. Louis, St. Louis, Missouri, United States of America; 3 Max Planck Institute for Ornithology, ‘Vogelwarte Radolfzell’, Radolfzell, Germany; 4 WildCare Institute, Saint Louis Zoo, St. Louis, Missouri, United States of America; 5 Muséum National d'Histoire Naturelle, Ménagerie du Jardin des Plantes, Laboratoire de Conservation des Espèces, UMR 5173 Muséum-CNRS, 57, Paris, France; 6 The Durrell Institute of Conservation and Ecology, The University of Kent, Canterbury, Kent, United Kingdom; 7 Save the Elephants, Langata Link Complex, Langata, Nairobi, Kenya; 8 Department of Zoology, University of Oxford, Oxford, United Kingdom; Monterey Bay Aquarium Research Institute, United States of America

## Abstract

A dramatic expansion of road building is underway in the Congo Basin fuelled by private enterprise, international aid, and government aspirations. Among the great wilderness areas on earth, the Congo Basin is outstanding for its high biodiversity, particularly mobile megafauna including forest elephants (*Loxodonta africana cyclotis*). The abundance of many mammal species in the Basin increases with distance from roads due to hunting pressure, but the impacts of road proliferation on the movements of individuals are unknown. We investigated the ranging behaviour of forest elephants in relation to roads and roadless wilderness by fitting GPS telemetry collars onto a sample of 28 forest elephants living in six priority conservation areas. We show that the size of roadless wilderness is a strong determinant of home range size in this species. Though our study sites included the largest wilderness areas in central African forests, none of 4 home range metrics we calculated, including core area, tended toward an asymptote with increasing wilderness size, suggesting that uninhibited ranging in forest elephants no longer exists. Furthermore we show that roads outside protected areas which are not protected from hunting are a formidable barrier to movement while roads inside protected areas are not. Only 1 elephant from our sample crossed an unprotected road. During crossings her mean speed increased 14-fold compared to normal movements. Forest elephants are increasingly confined and constrained by roads across the Congo Basin which is reducing effective habitat availability and isolating populations, significantly threatening long term conservation efforts. If the current road development trajectory continues, forest wildernesses and the forest elephants they contain will collapse.

## Introduction

Roadless wilderness is widely recognised for its high conservation value in terrestrial ecosystems [1], and therefore its preservation is frequently a primary goal of conservationists [2]. Conversely, developers and economists value roads as a cornerstone of efficient natural resource exploitation, and a prerequisite of economic development and poverty alleviation [3]. Roads improve access which facilitates resource extraction, human settlement and habitat degradation [4]. They fragment once contiguous habitats into smaller, isolated patches triggering numerous negative ecological consequences [5]. As road developments expand across the globe, understanding their impacts on ecosystems, populations, and individuals is increasingly important if ecologists, economists, and developers are to mitigate their negative ecological impacts while maintaining their socio-economic importance [2], [6].

The Congo Basin is among the world's great wilderness areas [7] containing the second largest rainforest block on earth, outstanding biological diversity, and its full complement of post-Pleistocene megafauna, including great apes and the forest elephant (*Loxodonta africana cyclotis*) [8]. It also contains globally important reserves of high quality timber, minerals, and other natural resources, critical to the economies of central African states [8]. Industrial-scale commercial exploitation of these natural resources is expanding dramatically and access to them relies on the proliferation of a network of public and private roads and rail infrastructure to supplement river transport[9].

International conservation efforts managed through the Congo Basin Forest Partnership (CBFP) focus on sustainable ecosystem management and the maintenance of wilderness and viable populations of apes, elephants, and other megavertebrates in 12 “conservation landscapes” centred around a network of protected areas [10]. Management of these landscapes is intended to promote sustainable natural resource exploitation practices to ensure the ecological integrity of priority areas of biodiversity conservation including the national park network. Landscape scale management is an important conservation tool since protected areas alone are frequently too small to ensure the survival of viable populations of large-bodied, wide-ranging species such as top carnivores [11], and savannah elephants [12], which routinely travel outside park borders [13] into unprotected areas. In the Congo Basin, forest elephant density in and around protected areas is determined by the area of roadless wilderness rather than the size of protected areas since illegal killing is concentrated close to roads [14], particularly those that are afforded no protection [15].

As road infrastructure advances into remaining wilderness, forest elephants face a choice between two broad behavioural responses; reduce their home range size and become increasingly restricted in order to avoid roads (termed a *siege* strategy here), or continue to range widely, crossing roads despite the risk of mortality (a *skirmish* strategy). Both courses of action are detrimental to survival. It has been shown that home range restriction by fencing in savannah elephants may lead to local overpopulation, increased local feeding intensity and habitat degradation [16], while continued wide ranging in the face of human encroachment may dramatically increase direct mortality from illegal killing [14].

In this paper we use the first GPS telemetry data from forest elephants to evaluate which of our proposed strategies, “siege” or “skirmish”, is adopted by forest elephants in response to roads by examining two questions: 1) does the size of roadless wilderness influence the size of forest elephant home range? and 2) do forest elephants cross roads and if so do they differentiate between roads inside and outside of protected areas? We then discuss the impact of recent and current road encroachment in wilderness areas, and finally, we propose two management actions to mitigate the impact of roads on elephants and other mobile terrestrial species in the Congo Basin.

## Results

### Roadless wilderness and forest elephant home range

**Figure 1:**
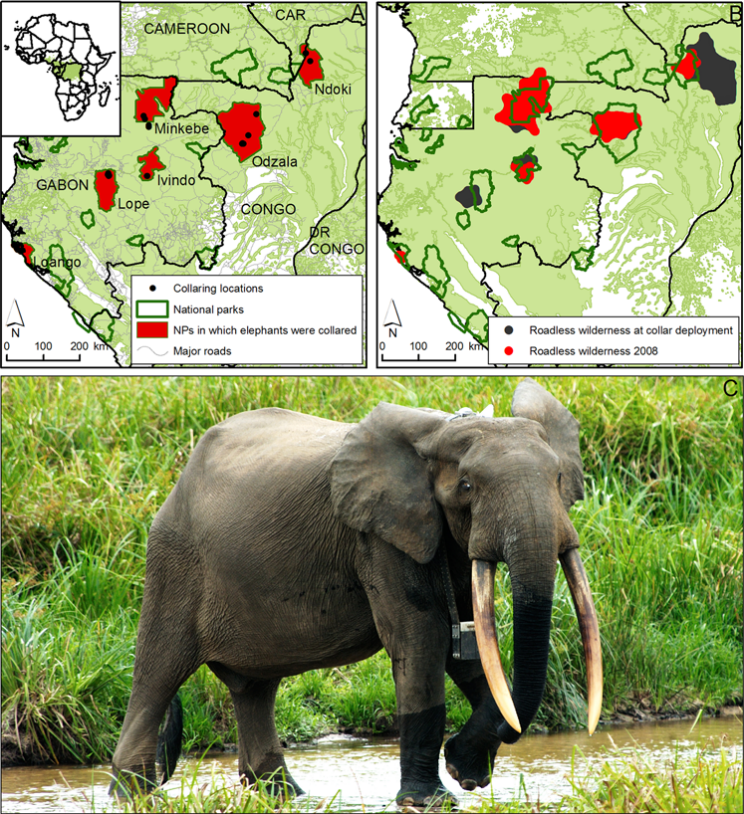
Study area, illustrating a, National parks in which collars were deployed and collaring locations. b, Change in roadless wilderness size collar deployment dates to the present. Note that total wilderness at the time of collar deployment is represented by red and black areas combined, while red colour alone represents wilderness remaining in 2008 c, Bull forest elephant in Ivindo NP fitted with a GPS telemetry collar.

We fitted GPS telemetry collars to a total of 28 forest elephants living in six roadless wilderness areas located in priority conservation areas [10] in Congo, Central African Republic, and Gabon. The size of roadless wilderness ranged from 49 km^2^ at the Loango site to 11,793 km^2^ at the Ndoki site (Table 1, Figure 1a,b,c). We estimated four metrics to characterise forest elephant home range: Minimum Convex Polygon (MCP) size, Maximum Linear Displacement (MLD) defined as the longest axis of the MCP, 95% Fixed Kernel Home Range (95%KHR) size, and 50% Fixed Kernel Home Range (50%KHR) size which we equated with the core home range area.

**Table 1:** Basic ranging statistics for collared forest elephants. Note that values in all but the last column were generated from the raw collar data, while values in the final column (PRC) were the result of simulated trajectories (see Materials and Methods for details).

Name	Sex	Site	Start and End dates (monthyear)	N days collared	Roadless polygon (km^−2^)	MCP^1^ (km^2^)	MLD^2^ (km)	95% KHR^3^ (km^2^)	50% KHR^4^ (km^2^)	PRC^5^
Kumu	F	Ndoki	0101-0302	415	11,793	1,570.3	57.4	346.3	42.0	51.9
Sparkey	M	Ndoki	0101-0601	130	11,793	1,117.9	104.1	461.3	59.4	15.4
Spikey	F	Ndoki	0101-1101	297	11,793	2,226.3	98.3	2,402.1	272.5	62.7
Sue	M	Ndoki	1000-0701	259	11,793	676.8	41.0	211.2	13.9	39.4
Chloe	F	Ndoki	1098-0199	60	11,793	824.8	59.9			
Annelisa	F	Lope	0703-0804	390	1,152	36.9	8.7	8.4	0.7	83.4
Ernestine	F	Lope	0703-1104	489	1,152	555.6	41.1	161.2	15.3	94.7
Iona	F	Lope	0703-0205	573	1,152	957.3	44.8	539.6	45.7	97.2
Nuri	F	Lope	0703-0204	61	1,152	247.2	24.9	220.2	27.6	79.1
Africa	F	Ivindo	0803-0205	619	1,578	406.1	40.2	158.6	18.2	57.1
Ella	F	Ivindo	08-3-0205	560	1,578	496.8	38.1	164.9	18.4	66.3
Pfeffer	M	Ivindo	08-3-0205	569	1,578	324.4	30.7	40.7	8.0	76.8
Powel	M	Ivindo	0803-0804	383	1,578	1,265.5	52.8	106.7	26.6	74.3
Bambala	F	Loango	1103-0304	121	59	25.9	10.2	19.8	2.8	90.5
Iwolo	F	Loango	1203-0505	516	59	113.7	31.0	141.5	24.3	77.1
Lione	F	Loango	1103-0605	558	59	51.8	10.6	26.2	1.4	69.9
Malonge	F	Loango	1103-0505	541	59	52.7	14.9	12.8	2.5	78.3
Mireille	F	Loango	1203-1104	362	59	51.0	12.8	39.7	5.4	46.4
Nanou	F	Loango	1203-0705	587	59	158.3	29.9	92.9	12.1	78.1
Dieudonne	M	Odzala	0604-0305	270	4,896	291.5	31.7	267.5	22.3	39.4
Lango	F	Odzala	0604-0505	334	4,896	346.7	25.0	243.9	16.1	78.7
Moba	F	Odzala	0604-0505	334	4,896	119.0	17.4	31.7	5.5	14.1
Mouadje	F	Odzala	0604-0805	430	4,896	811.9	81.5	97.6	27.6	81.7
Tommy	M	Odzala	0604-0805	436	4,896	309.6	25.0	7.7	1.5	15.0
Madame Nguendi	F	Minkébé	0107-0108	351	9,514	319.0	38.9	158.4	29.0	34.2
Mossimbo	M	Minkébé	0107-0807	192	9,514	814.2	64.9	138.0	46.9	10.2
Mwasi a Mossimbo	F	Minkébé	0107-1007	260	9,514	36.2	8.6	19.8	1.7	0.0
Sharon	F	Minkébé	0107-1007	258	9,514	1,103.3	82.6	1,011.1	105.7	20.9
Mean				372.9	4,742.0	546.8	40.3	264.1	31.6	56.8
SD				154.2	4,566.0	539.2	26.7	477.8	53.3	29.1

1 Minimum Convex Polygon Home Range.

2 Maximum Linear Displacement.

3 95% Fixed Kernel Home Range.

4 50% Fixed Kernel Home Range.

5 Proportion of 1000 correlated random walks which crossed an unprotected road.

We used Generalized Additive Models (GAMs) [17] to investigate the relationship between 4 home range metrics and roadless wilderness area, and to assess the influence of several other covariates, including Number of days collared, Site (the protected areas in which the individuals were collared), Sex and Landscape (landscape here refers to the priority conservation areas in which the protected areas were embedded) (Table 1). Only roadless wilderness area was consistently statistically significant in all the models, explaining 51.7% (MCP), 38.9% (MLD), 36.1% (95%KHR), and 28.5% (50%KHR) of the deviance in the final models for each of those metrics which included only that covariate. Of the remaining covariates, only Site was significant for the models with response MCP size and MLD (however, the Generalized Cross Validation score indicated that the Site variable should not be included). Including Number of days collared as an offset variable gave improved models for the responses MCP size and 95%KHR size (see supporting information for details of the model diagnostics).

**Figure 2:**
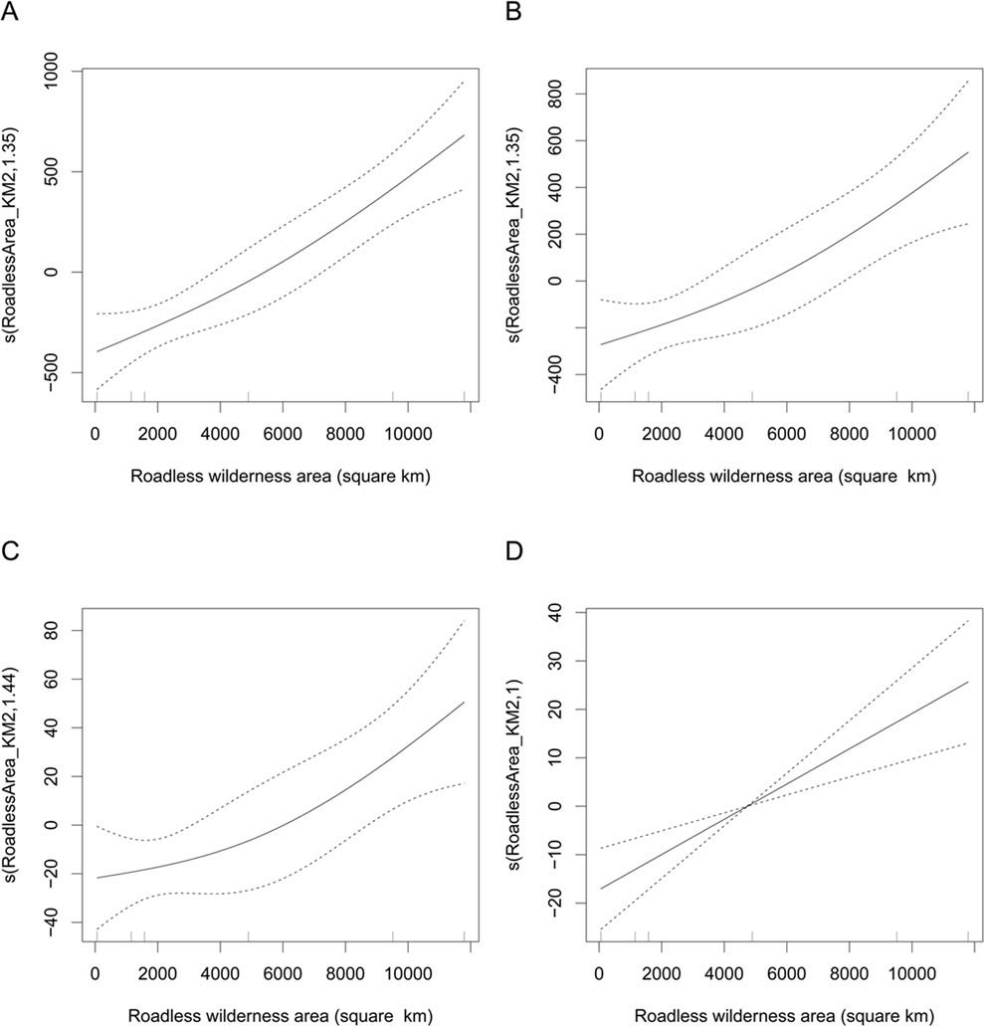
Estimated Conditional Dependence of a, MCP size. b, 95%KHR size. c, 50% KHR size. d, MLD on roadless wilderness area. Estimates (solid lines) and confidence intervals (dashed lines), with a rug plot that displays a vertical line for each data point along the x-axis of the plot, are shown. To avoid over-fitting, the degrees of freedom for these models were restricted to 2.

We found a positive relationship between the estimated conditional dependence of the four response variables and the size of roadless wilderness (Figure 2). The relationship is very similar for the first 3 response variables while MLD increases linearly with roadless wilderness area. Thus not only did roadless polygon area influence the extremes of ranging behaviour (MCP and MLD) and normal patterns of movement (as indicated by the 95%KHR which is widely thought to exclude exploratory movements and areas that an animal will never visit again [18]) but it also strongly affected the size of forest elephant core areas.

### Road Crossing Behaviour

Collared forest elephants routinely crossed protected roads that were located inside protected areas. The four elephants collared in Minkébé NP did not have the option to cross protected roads since they did not exist at their site, but of the remaining 24 collared elephants, 17 individuals crossed protected roads at least once, and many crossed on tens of occasions. By contrast only one individual called Mouadje, a female with dependent offspring, crossed an unprotected road outside of a protected area, a striking result given an accumulated total time of 28.5 years of telemetry data from 27 elephants (we excluded one elephant, Chloe, from this analysis due to poor data quality). We investigated the potential that each elephant had to cross unprotected roads by generating 1,000 correlated random walks (CRW) and calculated the proportion that crossed an unprotected road at least once (Table 1 and Figures S7, S8, S9, S10, S11, S12). The results of the simulations showed that all but one of the elephants had the potential to cross unprotected roads, in comparison to only one which actually did so. The variation in the proportion of CRWs that crossed roads was influenced by both the movement characteristics of each individual elephant and the spatial distribution of the unprotected roads in its neighbourhood.

**Figure 3:**
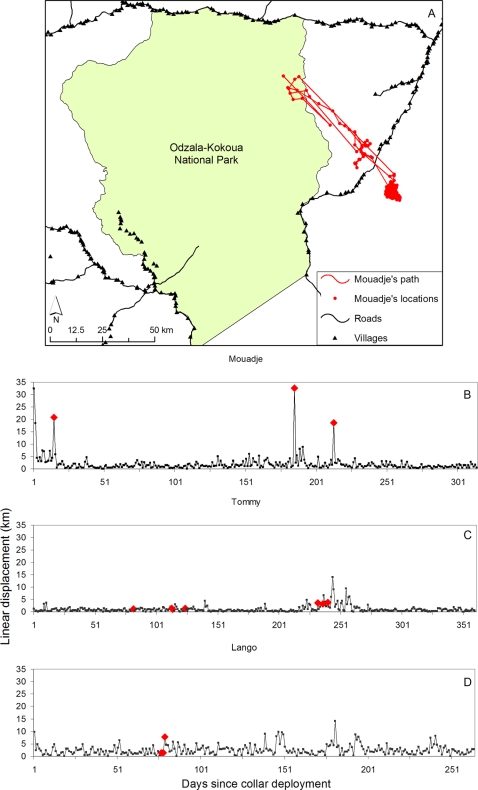
Road crossing by elephants at the Odzala site. a, Mouadje's trajectory including the 3 road crossing locations. The area of highest occupancy outside the national park was centred on a large area of open canopy Marantaceae Forest [36], dominated by a dense understorey of *Megaphrynium macrostachyum* (Marantaceae) a major food source for forest elephants that sometimes originates as a result of past human disturbance [37], which is also abundant in the Odzala NP. Mouadje's long trajectory which took her across the road was probably driven by the desire to access mineral deposits in the park at the Mouadje Bai b, Mouadje's daily travel distances, with the three unprotected road crossings marked by red diamonds. c&d, Daily travel distances for Tommy and Lango with protected road crossings marked by red diamonds.

The characteristics of Mouadje's road crossings are spectacular. On the three occasions when she traversed a road while collared, she crossed at the point furthest from any village in her potential range, which included 133 inter-village intervals over a total road distance of ca. 250 km (Figure 3a). Her mean daily travel distance during normal movements was 1.7 kmday^−1^ (SD 2.4), but when crossing an unprotected road this increased over 14 fold to a mean of 24.1 kmday^−1^ (SD 7.5) (Figure 3b). At the same site (Odzala) two other collared elephants crossed a protected road within the national park on 11 occasions, however travel speed was not appreciably different than for movements which did not involve a protected road crossing (Figure 3c,d). Tommy and Yango's mean speeds when crossing protected roads were 1.9 (SD 1.0) and 3.5 (SD 3.7) kmday^−1^ compared to 1.0 (SD 1.9) and 2.5 (SD 2.0) for other movements. While these data hint of an increase in speed during protected road crossings they are within the norms of daily travel speed of elephants across sites.

## Discussion

We conclude that forest elephants adopt a siege strategy in the face of road encroachment, rather than face the dangers associated with skirmishing. The overwhelming importance of a single variable, size of roadless wilderness, in defining home range size in forest elephants is impressive since the human footprint stamp on the telemetry sites is vastly more complex than our simplistic definition of wilderness [7], the inter-site ecology is highly variable, and elephants are an extremely adaptable species [19]. *A priori*, we had expected a sigmoid curve to describe the relationship between roadless wilderness size and home range size metrics, with home range reaching an asymptote as roadless wilderness size increased beyond a threshold after which ecological/physiological constraints alone would define home range characteristics. However, none of the 4 metrics show any tendency toward an asymptote over the range of wilderness size in this study, even though the Ndoki and Minkébé wildernesses were the largest *terra firma* roadless spaces in the entire Congo Basin at the time of collar deployment. Our study indicates that unconfined forest elephants may no longer exist anywhere in Africa.

The siege strategy may reduce the risk of mortality from poaching compared to skirmishing, but as roadless space decreases, siege will likely result in loss of access to widespread food resources, reduced dietary quality and increased feeding competition promoting aggressive social interactions with negative consequences for social cohesion and reproductive success which ultimately reduces population size [20]. The siege strategy may also increase the isolation of small sub-populations and reduce the genetic fitness and health status of small populations potentially increasing in the probability of extinction [21]. Finally, the restricted movements of besieged elephants may increase the destructive impacts of over-browsing on local vegetation [22], and reduce the effectiveness of forest elephant mediated seed-dispersal [23].

As roads in and of themselves are not a physical barrier to movement, forest elephants may avoid roads for 2 principal reasons; 1) roads may reduce resource quantity and quality, 2) proximity to roads may represent an unacceptable risk to elephants. The first can be discounted since in the absence of human threats forest elephants can occur at higher densities in logged and secondary forest than in primary forest due to an abundance of preferred herbaceous food plants [24]. Risk avoidance is the most compelling reason. Savannah elephants are known to perceive risk and avoid exposure to it [25], chimpanzees alter their behaviour depending on their risk assessment of forest roads [26], as do less intelligent species such as moose [27]. Even in the depths of the Congo forest the ranging behaviour of the largest extant terrestrial mammal is driven by fear.

Since the telemetry data were collected, construction of unprotected roads has caused an enormous loss of roadless wilderness area to 3 of the 6 telemetry sites (Figure 1b). The Ndoki wilderness has shrunk by over 11,000 km^2^ (−88.9%) in 8 years, the Ivindo wilderness by 940 km^2^ (−59.6%) in 5 years, and the Lopé wilderness has disappeared within the last 5 years. In all cases the roads were built to facilitate extractive industry, and were accompanied by significant additional infrastructure including logging towns, sawmills, and in the case of Ivindo, a hydroelectric power station and accommodation for an estimated 2000 construction workers and associated infrastructure (roads, clinics, etc.) will likely be built in the heart of Ivindo NP, as one component of the massive Belinga Iron Mining Project. This project will further affect regional elephant ranging through the construction of additional roads, local housing, a railroad and power transmission network, and will attract thousands of immigrants seeking employment or to provide diverse services and products, including bush meat and ivory. Two other regional national parks (Minkébé and Mwagne) are indirectly threatened by the potential impacts of the Belinga Project.

These are not isolated cases. The strategy of both the private sector and the international aid community in Africa is focussed on heavy investment in infrastructure, particularly roads [28]. Tens of billions of dollars are spent per year on roads to access timber, iron and other natural resources to supply global consumer demand, grow national economies and reduce poverty [29], [30], and meet the self-interest of donor nations [3]. Today, as regional development planning accelerates in the Congo Basin, human population density remains relatively low and several large tracts of wilderness still exist, and a last window of opportunity presents itself to implement a coherent road strategy that achieves an optimal balance between socio-economic development goals, efficient natural resource extraction, and the preservation of large wilderness areas.

To maintain viable elephant populations composed of individuals whose ranging behaviour is driven by ecological constraints rather than by fear, at least two management policies must be included and implemented in such a strategy. The first is to stop new road encroachment into remaining large roadless wildernesses, and the second is to reduce the factors that promote elephant's fear of roads. No new permanent roads should be built either around the peripheries of priority elephant conservation areas or penetrating deeper into them. To access natural resources that occur in or near to these areas, temporary exploitation roads should be built from the permanent road network radially into the wilderness interior which can be closed when the resource is exhausted. Reducing fear associated with roads requires investment of money and political will in law enforcement for effective anti-poaching, preventing settlement, mitigation of human-elephant conflict, and sustainable livelihood development for forest people. Key areas that provide connectivity between priority elephant populations or that give elephants access to seasonally important resources such as large swamps should either remain roadless, or existing roads should be kept free of human settlement and policed to reduce rates of poaching. National government, international lending agencies and private industry which promote, build and finance the infrastructure necessary to extract resources must bear the responsibility for the costs of impact mitigation. The costs of wildlife protection are trivial compared to the investments being made in resource extraction and the profits they generate. For example, the Belinga iron mine project, 50 km from the Minkébé wilderness in northeast Gabon will require at least $3 billion of pre-production investment in infrastructure alone [30]. This single enterprise budget is 34 times greater than the annual investment required to effectively manage a network of protected areas throughout the entire Niger Delta/Congo Basin forest region [31]. If the development trajectory and management of infrastructure of the Congo Basin continues without immediately ameliorating their negative ecological consequences, the last forested wildernesses of Africa and the forest elephants living in them may disappear.

## Materials and Methods

Forest elephants were fitted with GPS collars under supervision from the Field Veterinary Program of the Wildlife Conservation Society following methods described by Blake et al. [32]. Roadless wilderness areas were calculated using the Spatial Analyst extension of ArcView 3.3 [33], based on data provided by Global Forest Watch (GFW) supplemented with local knowledge for some recently built road segments. A raster grid of “distance from nearest road” (DISTRD) values was generated across the entire study region with a cell size of 1 km^2^. Grid cells were selected at increasing DISTRD until a bounded polygon was produced at the site with the least contiguous road network (Ndoki) which occurred at DISTRD = 31 km. Roadless wilderness area was calculated at each site as the number of cells at DISTRD≥31 km, which provided a comparative metric of relative roadless wilderness area by site rather than the absolute area retained between the road network.

We estimated home range metrics from the GPS telemetry data using the Animal Movement extension [34] for ArcView 3.3. Minimum Convex Polygons were calculated using all location data for each elephant, while fixed KHR estimates (using LSCV to generate the smoothing parameter) were based on subsamples consisting of 1 random fix per day to reduce the bias of variable GPS fix regimes.

Generalized Additive Models [17] were used to investigate the relationship between home range metrics and roadless wilderness area, and to assess the influence of several other covariates, including Number of days collared, Site, Sex and Landscape. Given their flexibility GAMs are particularly suited to investigating relationships in ecological data. GAMs were fitted in R [35] using the mgcv package [16]. We initially assumed a Gaussian distribution for all models. The standard diagnostic plots (Normal Q-Q, residuals vs. linear predictor, histogram of residuals, response vs. fitted values) used in model selection and assessment of fit indicated that the models were consistently giving lower fitted values when these were compared to the response values (Figures S1 and S2). The high values of home range metrics for some individuals at the Ndoki site, which are in contrast to the metrics at other sites or within the same site, contribute to this problem. These same diagnostics indicate some problems with model fit for the models with 95% Kernel Home Range size and 50% Kernel Home Range size as the response variable (Figures S3 and S4). Alternative models with a Poisson distribution and log link give mixed results (Figures S5 and S6). Although the deviance explained increases to 41.8% for 95% Kernel Home Range size, the diagnostics only improve marginally, whereas for 50% Kernel Home Range size the diagnostics improved and the deviance explained increased to 44.7%.

To assess possible differences in movement characteristics of forest elephants in relation to road protection status, we classified roads as either “unprotected” or “protected”. Unprotected roads were those roads in our dataset outside of protected areas, while protected roads were located inside protected areas. We did not have good information on either hunting levels or anti-poaching effort on the road system throughout our study areas, thus a more detailed assessment of the level of protection from hunting on roads was not possible. In the Loango, Lope and Odzala sites protected and unprotected roads were found in both dense forest and forest savannah mosaics, while in the remaining site all roads were in dense forest, with contiguous forest on both sides. The swathe of cleared vegetation created by the roads varied between 4 and 15 metres and all road beds were lateritic. Vehicle traffic was low on all roads throughout the region, with an estimated maximum of 20 vehicles per day (usually logging trucks) on the busiest sections. Data on actual traffic frequency by site or by road section are not available. Human habitation and the intensity of agriculture varied considerably across sites, though generally unprotected roads had associated settlement and small-scale slash and burn agricultural plots, while protected roads did not.

Low temporal resolution of the GPS data meant that we were unable to provide detailed quantitative analysis of movement behaviour of elephants when crossing protected roads. Correlated Random Walks were generated in ArcView using the Animal Movement extension. CRWs started at the arithmetic mean of the daily random fix data for each elephant. Daily displacement distances were resampled without replacement to preserve the individual movement characteristics and total path length of each elephant. We calculated a metric to reflect the potential of elephants to cross unprotected roads called Proportion of Road Crossings (PRC) (Table 1), as the proportion of 1000 randomly generated CRWs that crossed an unprotected road. To ensure biological reality the extent of the CRWs was limited to fall inside a circular buffer region of 104 km radius corresponding to the largest MLD recorded in the study, which biased our values towards underestimation of actual road crossing potential. The purpose of illustrating the unrestricted CRWs (Figures S7, S8, S9, S10, S11, S12) was to demonstrate how conservative the restricted CRWs were in evaluating road crossing potential.

## Supporting Information

Figure S1 Diagnostic plots for the Gaussian distribution model that included the Minimum Convex Polygon area as a response and the covariate roadless wilderness area with the number of days collared included as an offset value. To avoid over-fitting, the degrees of freedom for this model were restricted to 2.
(0.76 MB TIF)

Figure S2 Diagnostic plots for the Gaussian distribution model that included the Maximum Linear Displacement distance as a response and the covariate roadless wilderness area. To avoid over-fitting, the degrees of freedom for this model were restricted to 2
(0.75 MB TIF)

Figure S3 Diagnostic plots for the Gaussian distribution model that included the 95% Kernel Home Range area as a response and the covariate roadless wilderness area with the number of days collared included as an offset value. To avoid over-fitting, the degrees of freedom for this model were restricted to 2
(0.75 MB TIF)

Figure S4 Diagnostic plots for the Gaussian distribution model that included the 50% Kernel Home Range area as a response and the covariate roadless wilderness area. To avoid over-fitting, the degrees of freedom for this model were restricted to 2.
(0.32 MB TIF)

Figure S5 (a) Estimated Conditional Dependence of 95% Kernel Home Range area on roadless wilderness area. Estimates (solid lines) and confidence intervals (dashed lines), with a rug plot indicating observation density along the bottom of the plot, are shown. (b) Diagnostic plots for this Poisson distribution with a log link model. To avoid over-fitting, the degrees of freedom for this model were restricted to 2.
(0.56 MB TIF)

Figure S6 (a) Estimated Conditional Dependence of 50% Kernel Home Range area on roadless wilderness area. Estimates (solid lines) and confidence intervals (dashed lines), with a rug plot indicating observation density along the bottom of the plot, are shown. (b) Diagnostic plots for this Poisson distribution with a log link model. To avoid over-fitting, the degrees of freedom for this model were restricted to 2
(0.53 MB TIF)

Figure S7 Figures S7, S8, S9, S10, S11, S12 show a sample of correlated random walks (CRWs) showing raw elephant location data, unrestricted CRWs, CRWs restricted to 104 km from the arithmetic mean location of the MCP home range in relation to unprotected roads and national parks
(2.61 MB TIF)

Figure S8 Powel, collared in Ivindo NP, Gabon
(4.08 MB TIF)

Figure S9 Mouadje, collared in Odzala NP, Congo
(3.50 MB TIF)

Figure S10 Madame Nguendi, collared in Minkébé NP, Gabon
(2.99 MB TIF)

Figure S11 Mireille, collared in Loango NP, Gabon
(2.92 MB TIF)

Figure S12 Iona, collared in Lope NP, Gabon
(4.15 MB TIF)
