# Supplementary figures and images for: Roadless Wilderness Area Determines Forest Elephant Movements in the Congo Basin
Source: PLoS One. 2008 Oct 28;3(10):e3546. doi: 10.1371/journal.pone.0003546 (PMC2570334; doi:10.1371/journal.pone.0003546)

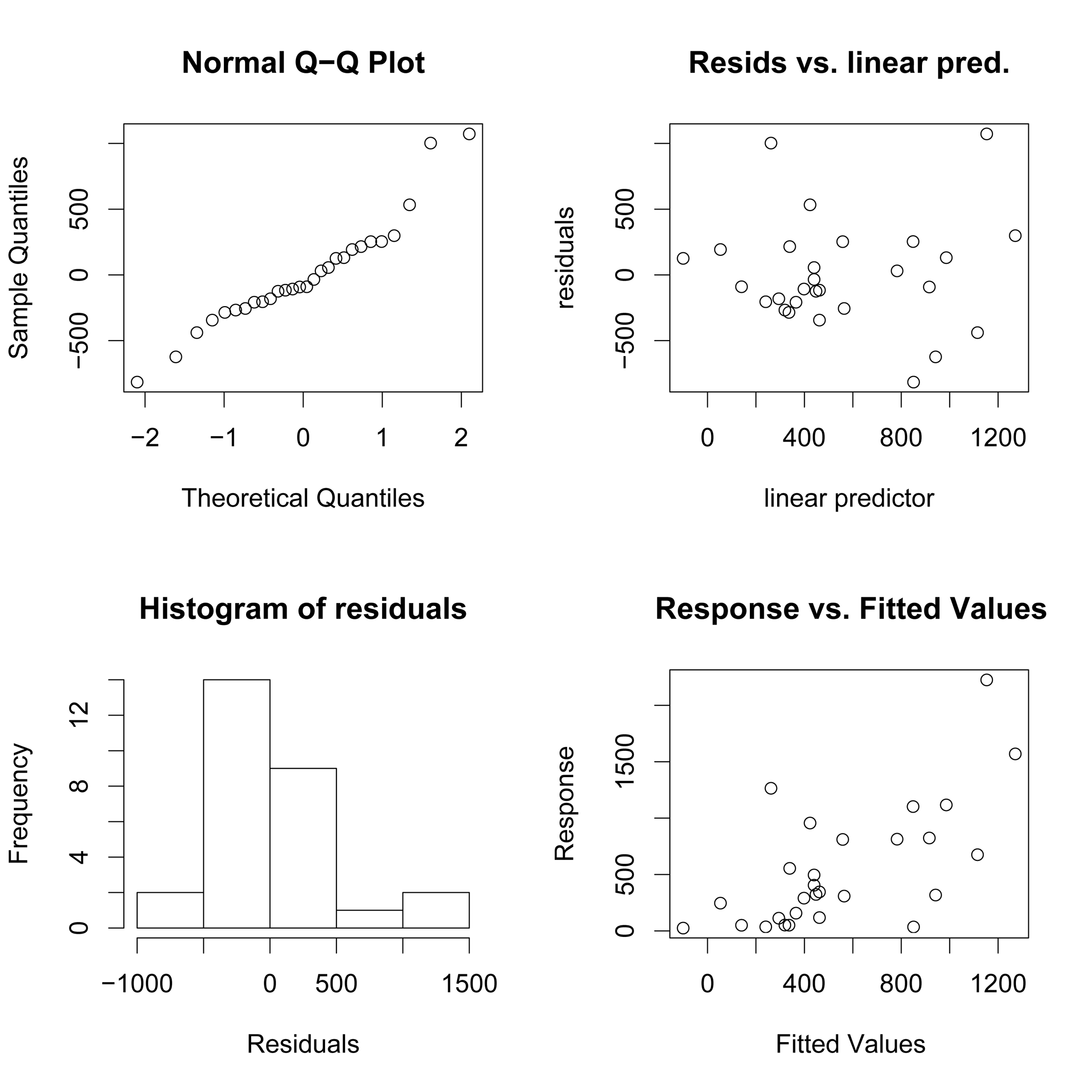

Supplement: Figure S1 — Diagnostic plots for the Gaussian distribution model that included the Minimum Convex Polygon area as a response and the covariate roadless wilderness area with the number of days collared included as an offset value. To avoid over-fitting, the degrees of freedom for this model were restricted to 2. (0.76 MB TIF) [file pone.0003546.s001.tif]

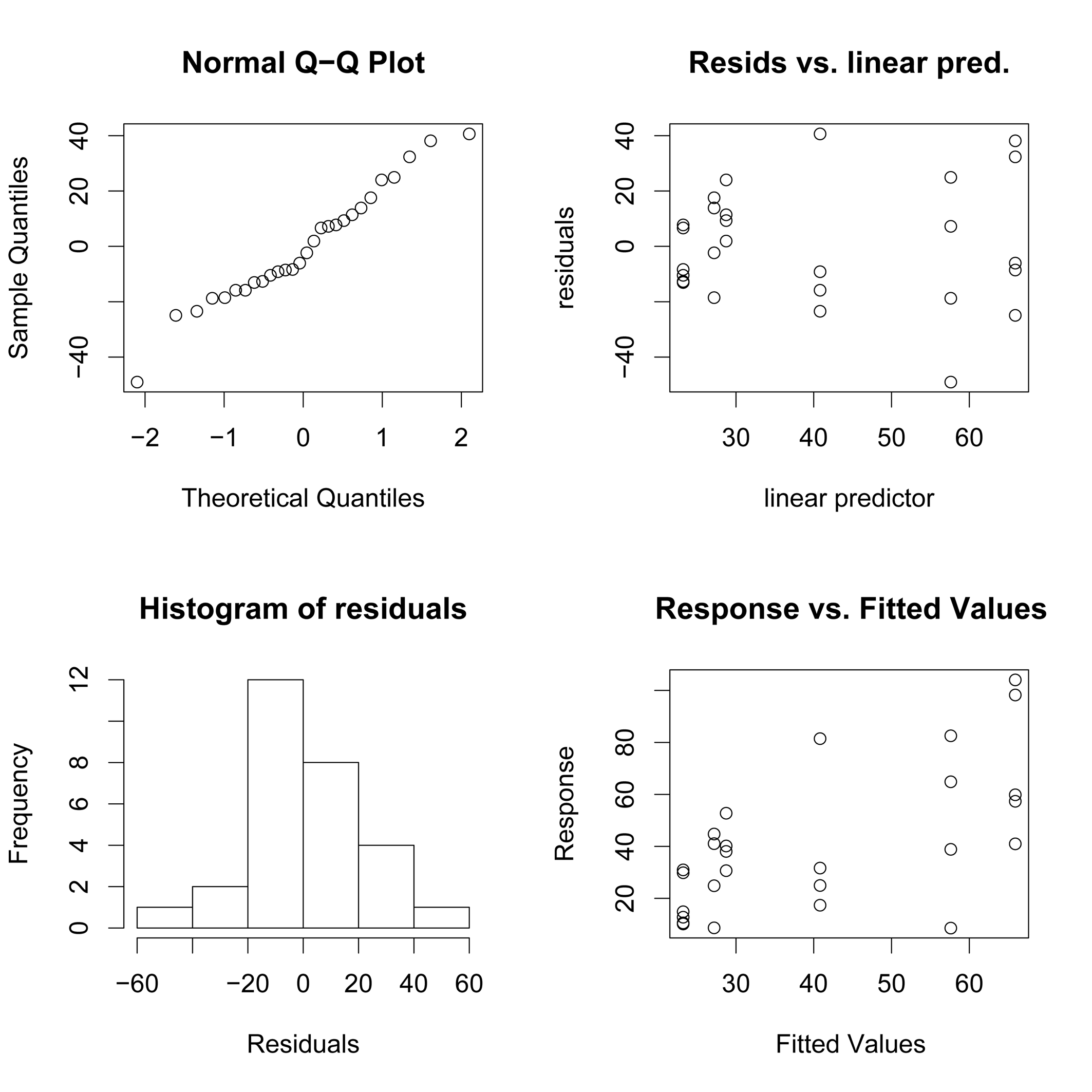

Supplement: Figure S2 — Diagnostic plots for the Gaussian distribution model that included the Maximum Linear Displacement distance as a response and the covariate roadless wilderness area. To avoid over-fitting, the degrees of freedom for this model were restricted to 2 (0.75 MB TIF) [file pone.0003546.s002.tif]

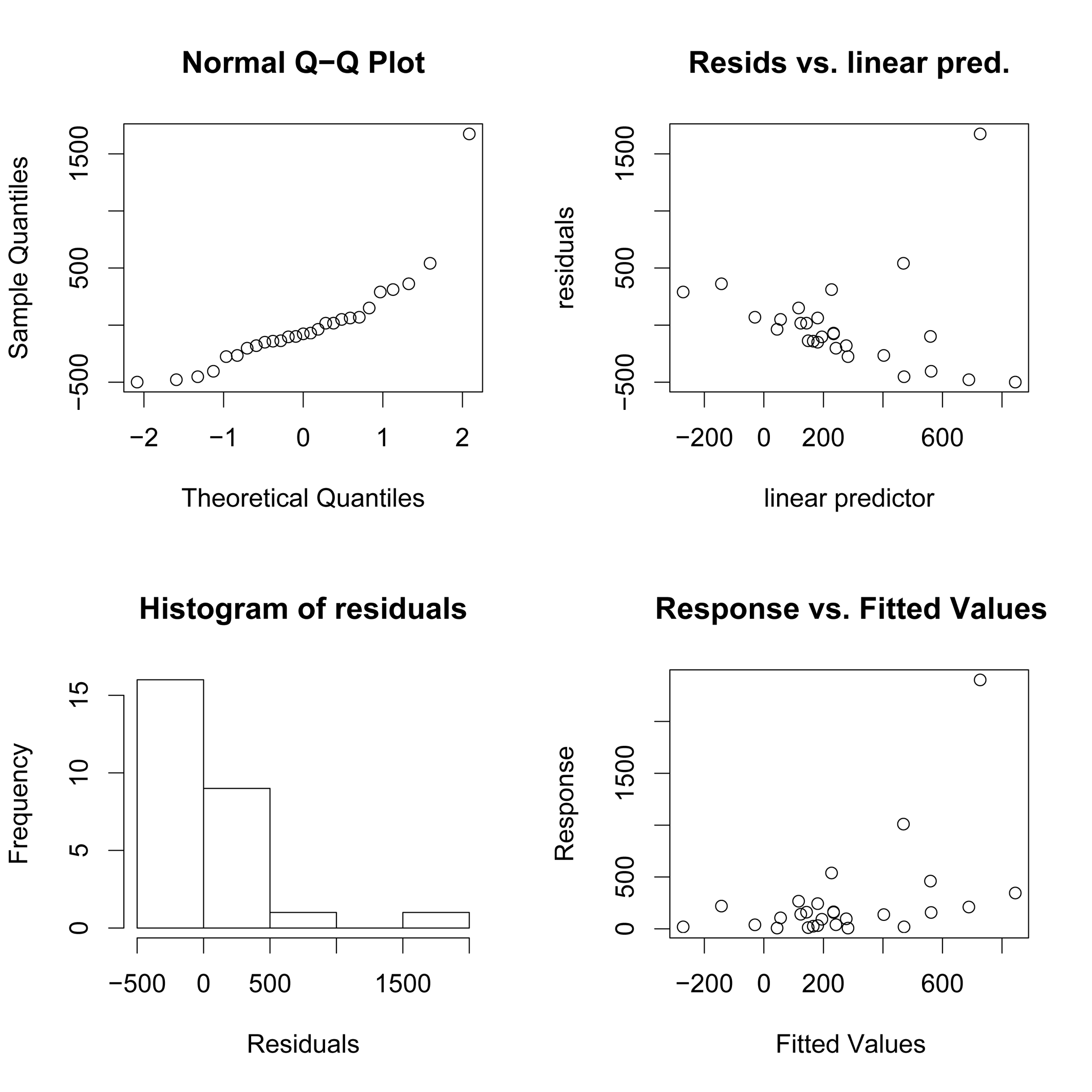

Supplement: Figure S3 — Diagnostic plots for the Gaussian distribution model that included the 95% Kernel Home Range area as a response and the covariate roadless wilderness area with the number of days collared included as an offset value. To avoid over-fitting, the degrees of freedom for this model were restricted to 2 (0.75 MB TIF) [file pone.0003546.s003.tif]

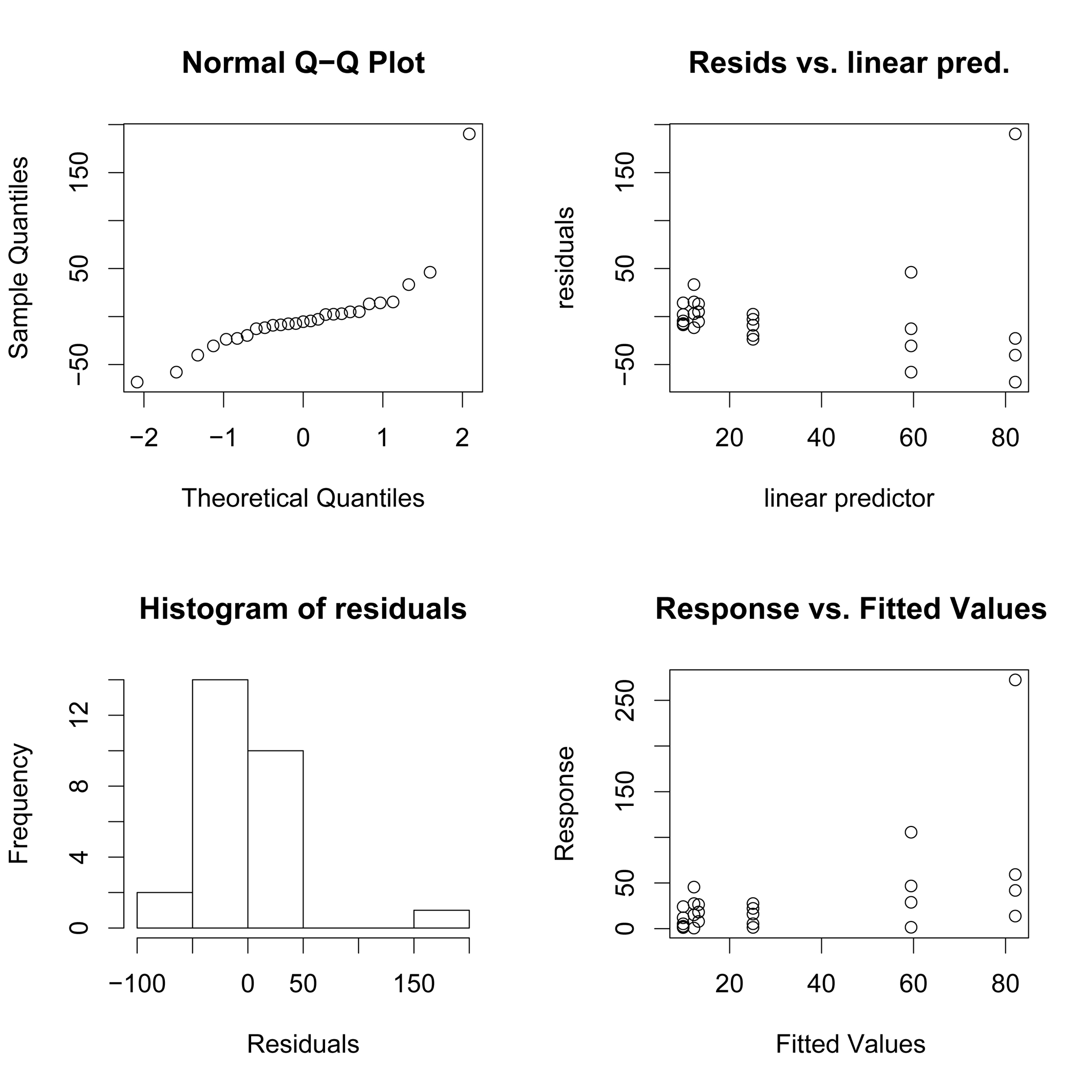

Supplement: Figure S4 — Diagnostic plots for the Gaussian distribution model that included the 50% Kernel Home Range area as a response and the covariate roadless wilderness area. To avoid over-fitting, the degrees of freedom for this model were restricted to 2. (0.32 MB TIF) [file pone.0003546.s004.tif]

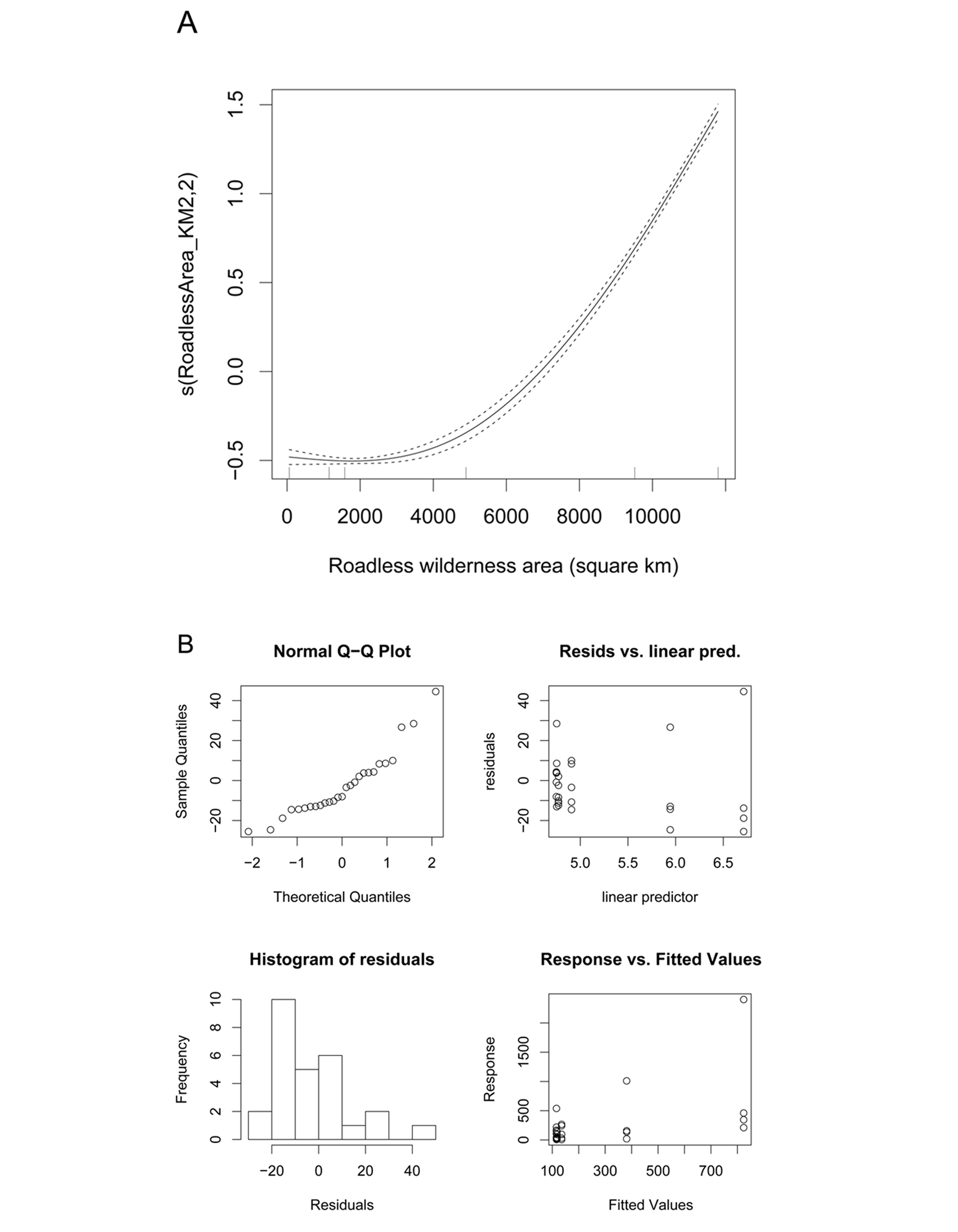

Supplement: Figure S5 — (a) Estimated Conditional Dependence of 95% Kernel Home Range area on roadless wilderness area. Estimates (solid lines) and confidence intervals (dashed lines), with a rug plot indicating observation density along the bottom of the plot, are shown. (b) Diagnostic plots for this Poisson distribution with a log link model. To avoid over-fitting, the degrees of freedom for this model were restricted to 2. (0.56 MB TIF) [file pone.0003546.s005.tif]

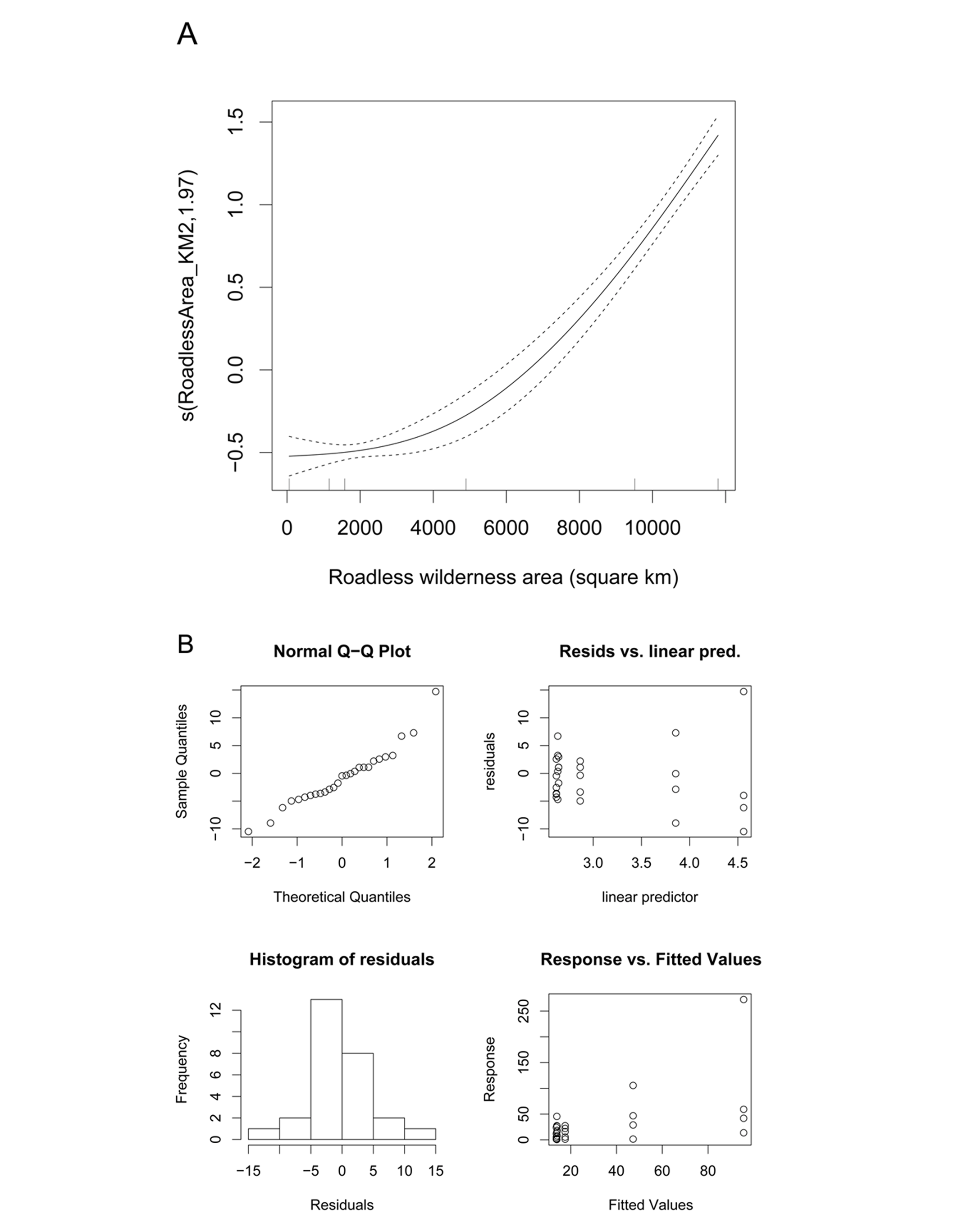

Supplement: Figure S6 — (a) Estimated Conditional Dependence of 50% Kernel Home Range area on roadless wilderness area. Estimates (solid lines) and confidence intervals (dashed lines), with a rug plot indicating observation density along the bottom of the plot, are shown. (b) Diagnostic plots for this Poisson distribution with a log link model. To avoid over-fitting, the degrees of freedom for this model were restricted to 2 (0.53 MB TIF) [file pone.0003546.s006.tif]

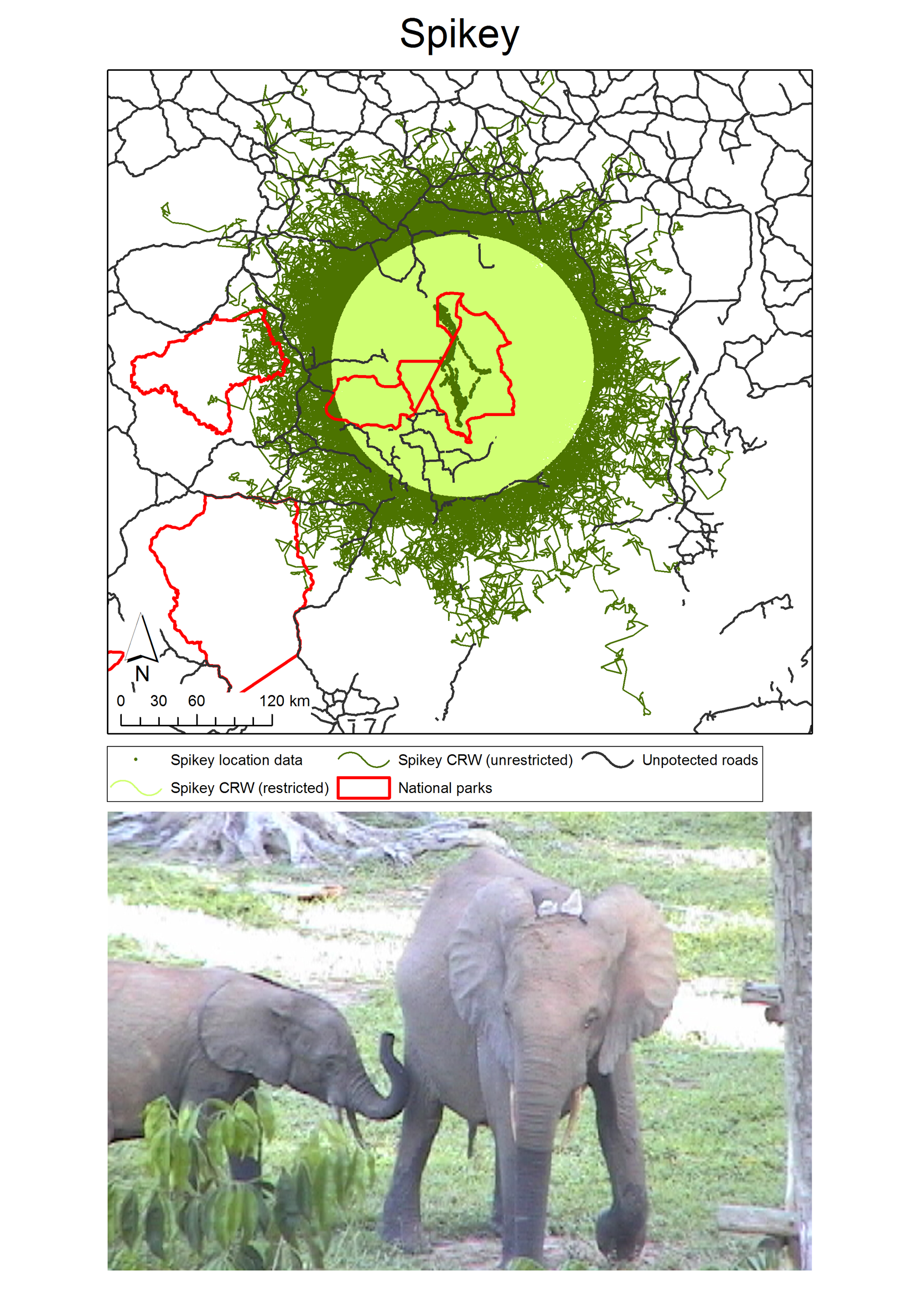

Supplement: Figure S7 — Figures S7, S8, S9, S10, S11, S12 show a sample of correlated random walks (CRWs) showing raw elephant location data, unrestricted CRWs, CRWs restricted to 104 km from the arithmetic mean location of the MCP home range in relation to unprotected roads and national parks (2.61 MB TIF) [file pone.0003546.s007.tif]

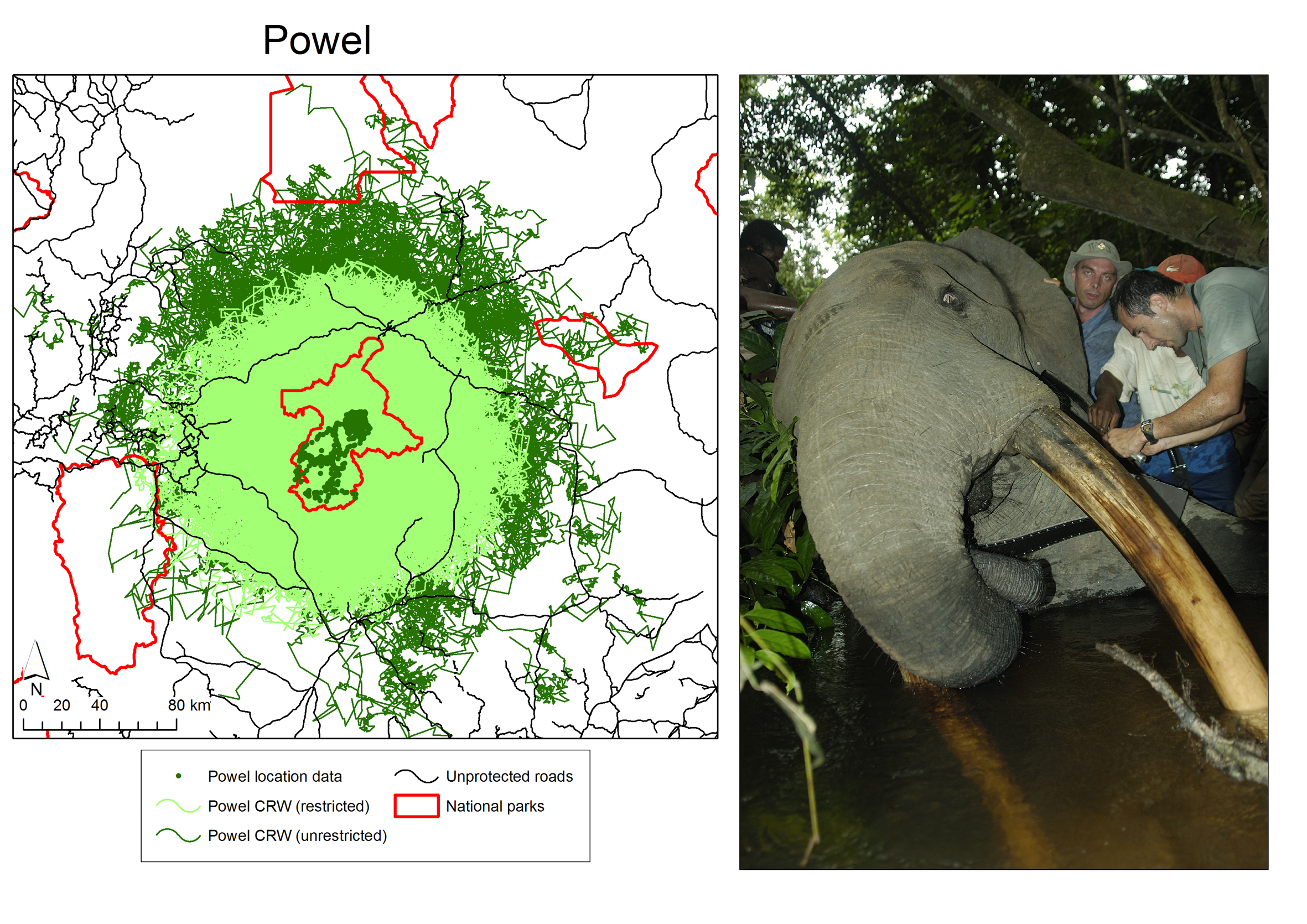

Supplement: Figure S8 — Powel, collared in Ivindo NP, Gabon (4.08 MB TIF) [file pone.0003546.s008.tif]

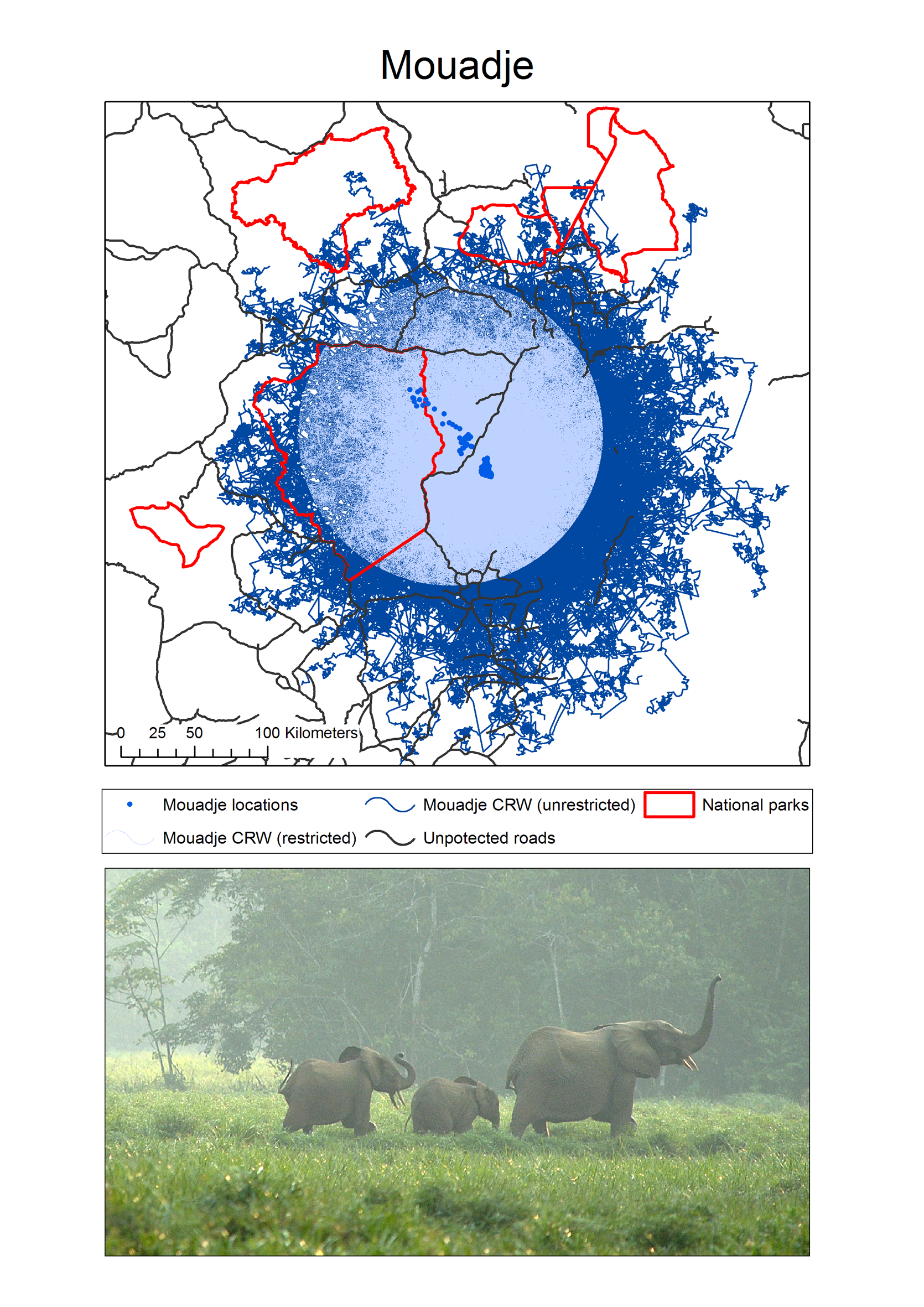

Supplement: Figure S9 — Mouadje, collared in Odzala NP, Congo (3.50 MB TIF) [file pone.0003546.s009.tif]

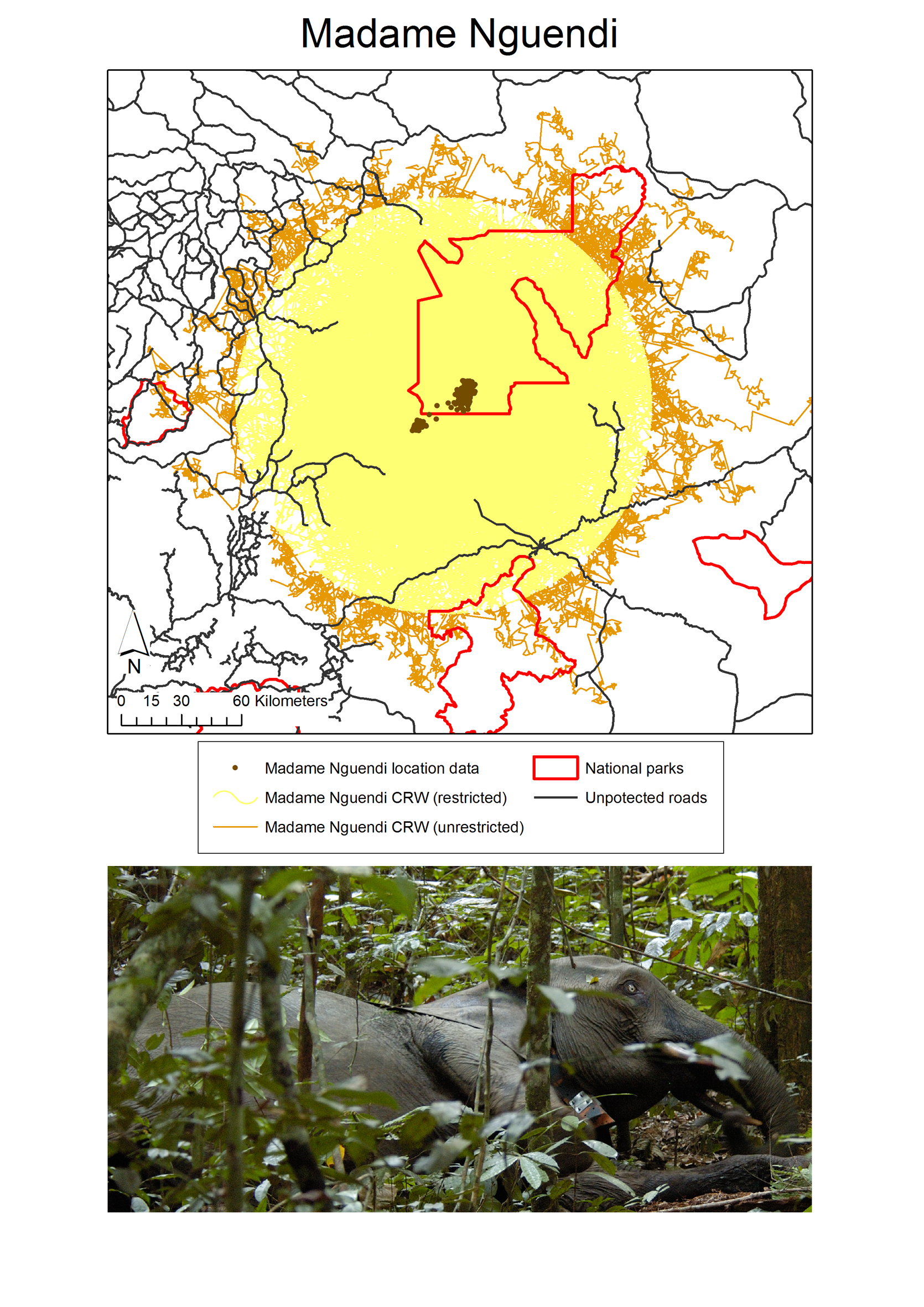

Supplement: Figure S10 — Madame Nguendi, collared in Minkébé NP, Gabon (2.99 MB TIF) [file pone.0003546.s010.tif]

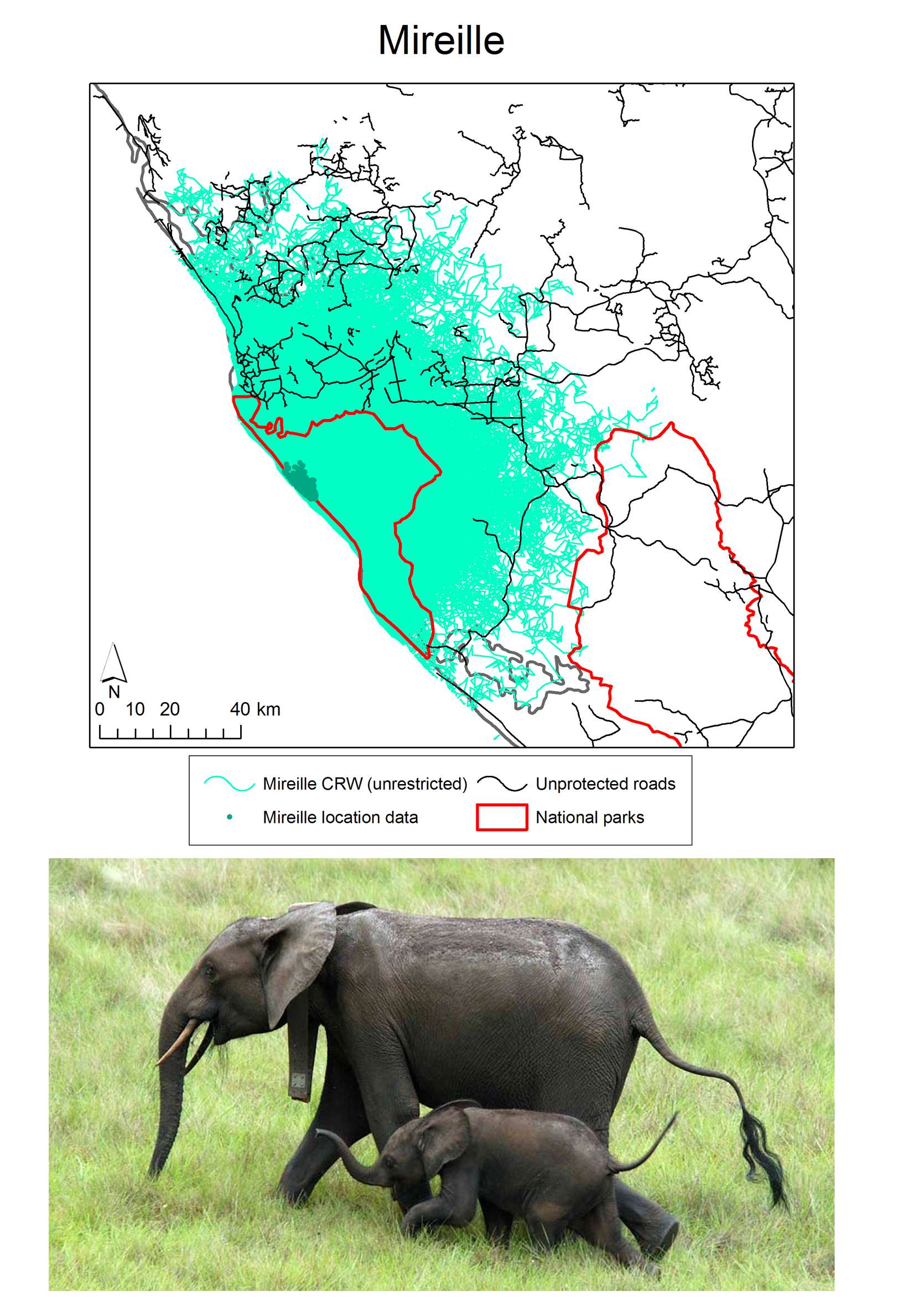

Supplement: Figure S11 — Mireille, collared in Loango NP, Gabon (2.92 MB TIF) [file pone.0003546.s011.tif]

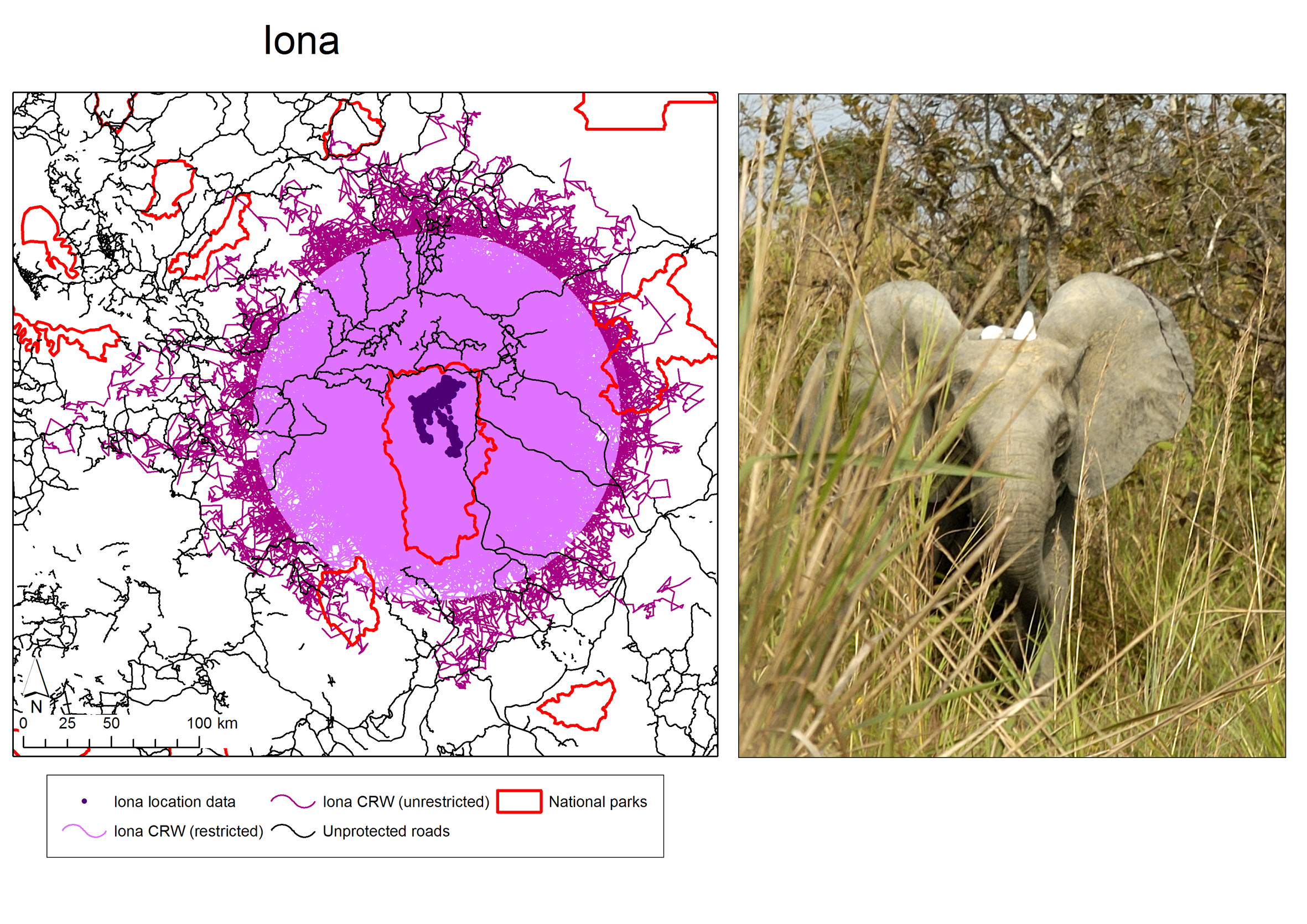

Supplement: Figure S12 — Iona, collared in Lope NP, Gabon (4.15 MB TIF) [file pone.0003546.s012.tif]
